# Medical and Physical Intelligence

**Published:** 1823-02

**Authors:** 


### MEDICAL AND PHYSICAL INTELLIGENCE.

1. *Some Account of the Medical Schools, &c. at Berlin.—(Extract of a Letter to* Dr. Granville, *from a Physician at Berlin.)*

[Continued from No. 286, page 539.]

There are two kinds of medical and chirurgical clinical instruction; the first fixed in the hospitals, the second ambulatory. Professor Hufeland, whose reputation is in exact relation to the distance from him, passes in Berlin in the rank of the first physicians; Dr. Heim, whose reputation travels in an inverse ratio to that of Hufeland, is, by the lower classes, hailed as the first, and, though he does not hold the highest situation in the opinion of the medical world, yet he is esteemed among the literati. He has caused himself to be drawn lately in a morning-gown, with a pipe in his mouth, and a night-cap on, the ordinary dress in which he gives his advice to the poor. He is a second Dubois. Hufeland holds himself higher, and presides at the Polyclinic School, or the *ambulatory clinic*, with much seriousness. This latter is a kind of dispensary, with this difference, that every patient is recommended by a young doctor, who goes to the patient's house when required, (which is very frequently,) sees the patient, and follows the plan of cure laid down by himself, if it is approved by Hufeland or his assistant, or some other plan laid down by them; he is obliged to give a daily account of the patient, and afterwards to recite the history.

Hufeland has raised in his own house an Academy of Medicine, of which Abernethy, Cooper, and others, were named honorary members. At this society I have heard a memoir read, in which the author mentions, with faith, the incredible story of the ejection of living frogs with the fæces. In this society the reading is not voluntary, each man reading something in his turn; and Hufeland is repaid for his trouble with a copy of the works that authors send him, or the honour of a dedication. I have attempted many times to learn what were the medical views of Hufeland, but I have not gathered much. I have seen, when the disease is well marked and evident, he then attacks it with energy enough, although he is timid in the doses of his remedies. Where the complaint is obscure, he is content to attack the symptoms, so that his prescription combines all the specifics for those symptoms,—such as opium against pain, calomel for bile, &c.

M. Berends, who enjoys a reputation in Berlin similar to Homberg of Gottingen, or Krukenberg at Halle, is at the head of clinical medicine, which is upon the same footing as the other clinical schools of Germany; that is, comprehending few sick, but those select cases; frequent interrogations and consultations with the pupils, who order and watch the prescriptions. Here, therefore, I have not found much to interest me: long prescriptions, tiresome stories, and questions interesting only to beginners; in short, the instruction is too much spun out.

The surgeon who enjoys the chief reputation is M. Gräfe, a quick, but *irascible*, operator. When he sees his patients, he sits in a corner about six paces from them, where he remains, and there is a young student who reports, either in writing or *viva voce*, on the state of the patient: so that he follows the same plan as the medical clinic, —interrogates, asks the opinion of his pupils, approves or proposes the method of cure. For my own part, I never met with any thing more tiresome than the repetition of these long and dry stories. In his amphitheatre, every thing is done with the greatest luxury and expense; and it is really wonderful to see how government accedes to every request of this strange reviver of ancient surgery, who, for the sake of saying that all is of his own invention, has changed every thing, and rejected the most approved models: as, for example, the amputating knife, of which I do not know a more ridiculous form than his; the convex is the cutting edge, and the whole weighs about a pound. I only mention this to show how he has changed every thing. He amputates fingers, as stone-cutters hew stones. I asked if the guillotine formed part of his surgical armoury, to cut off the large members at one blow? but they laughed at me, not understanding the point of my question.

According to these principles, we ought not to be surprised at finding the guillotine among the instruments of VALATANO, who regards the rapidity of his operations more than their consequences. I should have much to say concerning this person, who, that he may not dirty the orders with which he is decorated, and especially the cross which he carries suspended from his neck with a yellow riband, (a Russian decoration,) puts a piece of black cloth round his neck, a pair of pantaloons of the same colour, and a long morning-gown of the same materials: in fine, he looks like a necromancer; and employs eight or ten assistants when he performs an amputation. He is always in a passion, and things never go on according to his mind: in fact, he has no *sang froid.* There is, however, in his clinical department an institution which appears excellent, but which is not free from inconvenience, like all others. I allude to the practice of suffering the young men to operate, who, although well practised on the dead body, are not models to copy when they operate on the living. In fact, I have seen a young student operate for cataract, without the master putting his hand to it; whereas, all the rest were begun by pupils, and finished by the master. The arsenal (surgical) of Gräefe is very similar to that of Heister; that is, there are particular instruments for each operation. Here it is much esteemed; but a time will come when simplicity will please even at Berlin. Not only the ancient instruments and bandages are in use, but their Greek names are in great fashion with him.

Professor RUST, no less a chatterer than Gräfe, is another of the clinical lecturers (surgeons) at the Hospital of Charity, the only great civil hospital. He has passed much time at Vienna. He is author of a work on Coxalgia, ornamented with fine engravings, but containing nothing new. I have seen him employ the cautery with more pretension, and with a great parade of remedies, for he is a great enthusiast for medicine. I think that more money is spent in medicine than in the diet and surgical treatment both put together. The expense of apparatus for fractures is considerable, great luxury in the beds and in the machines, utensils, tables, &c. but the patients are crowded; and this is, in my opinion, the cause of the gangrene that rages here, as well as in many hospitals of Italy. There is in the hall a machine nearly similar to that of Hippocrates, for reducing luxations; and I have seen Rust fail in many attempts upon a recent luxation of the humerus, because his power was not properly directed, and his method of extension directly upwards, the arm extended upon the trunk, without any fixed counter-extension. This poor fellow, (the patient,) who was placed in every possible position, really excited my pity. Here is a ward for diseases of the eyes, and for the venereal disease. This first, like all the ophthalmic wards in Germany, is furnished with black doors, and curains of the same hue. The syphilitic patients are put under the *great*

*cure*, as Professor Rust calls the administration of mercury according to a particular formula of his own.

At the Charity, there is also a course of midwifery given by Professor KLUGE, who is a good man-midwife. Government furnishes money for the purchase of all the instruments, ancient and modern; and I believe there are not less than sixty kinds of forceps.

The hospital for lunatics is also in the Charity; and I only mention it to give you an idea of their occupations, which consist of cutting wood for the establishment, raising water, designing, digging, and filling up hollows for mere amusement, or some other thing equally insignificant. The room where they draw is really worthy of observation, and where these lunatics are so occupied and serious, that, to see them at work, and to judge from their performances, one would not take them to be insane. By this method, the great indication of withdrawing their minds from the causes of their disorder is fulfilled; and the result is really beneficial. Dr. NEWMANN procures models for them, and, if they do not know how to draw, there is a master, who is also a lunatic, and who teaches them.

The medical man who resides in the house is very well paid, but forbidden to see other patients, from the apprehension that he might be tempted to neglect the patients of the establishment.

[To be continued.]

---

2. *Remarkable Formation of the Organs of Generation.* [Extract of a Letter from the Chevalier ALBRECHT VON SHÖNBERG, M.D. residing at Naples, to the Editor of the Medicinish et Chirurgische Zeitung, 15th August, 1822.]

THE anomalous formations of the organs of animals have always excited the greatest interest, and received the attention of naturalists: on this account, much has been said and written, in every age, about monsters and the animals called hermaphrodites. Without, in the smallest degree, wishing to agitate at present the question relative to the formation of hermaphrodites, it may most certainly be considered as fully established that these formations are much more seldom met with in nature, the nearer the animal which shows them approaches to man. Thus, although we frequently meet with the most perfect specimens of hermaphroditism in imperfectly organized bodies, such as plants and several animals,—viz. in several species of worms, as in the snail,—still it is utterly unknown, at least to me, that any such perfect examples have been met with in the higher classes of animals, with the exception of several kinds of fishes, according to Cavolini, and, more lately, according to the researches of Rudolphi.

In the history and illustration of this point, I consider the following example as a most rare and peculiar one: it was shown me by the present secretary of the Royal Institute of this place, Professor Stellati, who has been so fortunate as to discover it. The animal was a goat, which the Professor first showed alive, and in perfect health, to the members of the Institute, to whom he also pointed out the following external peculiarities:

There was no trace whatever of a scrotum; there were, however, two testicles in the neighbourhood of the groin, under the common integuments. The penis was but imperfectly covered with a prepuce, and a little curved towards the tail of the animal. The opening of the urethra was discovered about an inch from the penis itself, which in this case was quite shut. There was no trace of any linea alba on the abdomen, nor the slightest appearance of a navel. The horns were entirely wanting, although the animal was already more than a year old.

The animal was afterwards killed, and then the following anomalous appearances were met with:—After the urethra had penetrated in one uniform passage about three inches from the external parts, it divided itself into two passages: the right, and most elevated, division lost itself in its junction with the urinary bladder; the left, and lowest, passage ended in a sac, where the arrangement of the fibres were quite different. Internally, this sac was completely covered with a mucous membrane, which formed an innumerable quantity of rugæ on its surface. It cannot at all be doubted but that this was the vagina. At the extremity of this sac was the uterus, different from the former, by being furnished with much stronger fibres and a firmer texture. As for the other parts of this organ, the uterus was of the same structure as it usually is in these animals. In place of fallopian tubes, there were found two canals, which at their commencement were very wide, but they became narrower and narrower by degrees, till at last they were lost in the cellular membrane attached to the sheath of the epididymis. The commencement of this passage is the same, in every respect, as that of the fallopian tubes from the horns of the uterus. The excreting ducts, which usually pass out from the epididymis, terminating in the vesiculæ seminales, were in the present example situated on the opposite side of the vagina. The form of the vesiculæ seminales was like that of irregular tubercles; and mercury, injected into them, readily passed into their cavities, since it easily communicated with both sides by means of the ductus deferens. The ovaria were quite wanting. The testicles were large, well formed, and properly developed.

The urinary organs showed also several peculiarities. Thus, the right kidney was situated higher than the left: on this account, there was found a slight variety in the situation of the emulging vessels. The right kidney had, besides, three other bodies attached to it, which might with justice be viewed in the light of accessory kidneys; the left kidney, on the other hand, was entirely wanting. Lastly, the insertion of the urethra into the bladder was not as it usually is.

I have perused with peculiar pleasure an interesting account of this animal, in a letter, in which the oral account of the goat-herd to the proprietor of the animal is thus expressed:—The herd was very much struck with this animal, which always appeared to be more lascivious than the others; he, however, did not know whether it was of the male or female species, as it constantly sought intercourse with the one and sometimes with the other, without, however, being ever able to consummate the act with either.—(*Medicinish & Chirurgische Zeitung.*)

### 3. *Action of Magnesia on Salep.*

M. Brandes, of Hoxter, has made some experiments on a compound which is formed when calcined magnesia is put into a solution of salep. When twenty grains of salep were dissolved in four ounces of distilled water, and thirty grains of pure magnesia were added, the whole became, after some hours, solid and jelly-like; and even after a month it had not assumed the least putrid smell. Carbonate of magnesia had the same effect, but in a smaller degree. Neither the white of eggs, nor tragacanth gum, a weak solution of isinglass, nor of starch, assumed, on addition of magnesia, a similar jelly-like appearance. The mucilage of quince-seeds deposited the magnesia with a granular appearance, and the solution seemed to have become more fluid. Neither lime nor white bole produced a similar effect upon the solution of salep. The jelly is insoluble in water, fat oils, oil of turpentine, alcohol, or a solution of caustic potash. Acids, principally sulphuric acid, dissolve it partly; the remainder is more bulky, and is opalescent.—(*Annals of Philosophy.*)

### 4. *On the Volatile Oil of Bitter Almonds as a Poison.* By M. Vogel, of Munich.

To deprive the oil obtained from bitter almonds by distillation of its hydro-cyanic acid, he agitated it with a concentrated solution of potash, and distilled it to dryness. The oil volatilized with the water, and the residuum in the retort, contained cyanide of potassium. To be certain that the oil was entirely deprived of all its hydro-cyanic acid, he distilled it anew with potash; but this time the residuum contained no cyanide of potassium.

The volatile oil of bitter almonds, thus purified, is without colour, and heavier than water. Its taste is extremely acrid and burning; it crystallizes rapidly by contact of air; it dissolves easily in alcohol and ether, but only in very small quantity in water. The flame of its combustion is very brilliant, and accompanied with much smoke.

In order to find whether this oil, freed from its hydro-cyanic acid, was still poisonous, M. Vogel put a drop of it on the tongue of a sparrow: it died, after violent convulsions, in a few seconds. He poisoned a dog, two months old, with four drops of it. He hence infers that this volatile oil, well purified, produces on animals deleterious effects, analogous to those of the hydro-cyanic acid, although in a feebler degree. —(*Journal of Science.*)

### 5. *Proper State of Prussic Acid for Medicinal Use.*

A series of experiments has been undertaken by a company of associated physicians, surgeons, and naturalists, at Florence, to determine the best state of the hydro-cyanic or prussic acid for medicinal purposes. The experiments were made with great care, and varied several ways. Different preparations of the substance were used, rabbits being the animals on which they were tried. Their joint opinion is expressed as follows: "We may then conclude, from our researches, that the essential oil of the prunus laura cerasus is to be preferred in medical practice to all other preparations which contain the hydro-cyanic acid; for, unlike the distilled water of the plant, and pure prussic acid, it contains the same proportion of the acid, and is of the same power whether recently prepared or old, when made in one place or another, after exposure to the air, to light, or to heat. We think also that the oil of olives, or of almonds, is the most proper vehicle, in the proportion of an ounce to twelve drops of the essence; or in a smaller dose when employed by friction externally."—*(Journal of Science.)*

### 6. *New Kind of Truss.*

A TRUSS of a very ingenious construction has lately been submitted to our inspection, backed by a strong recommendation from a medical friend, who has proved its efficacy in his own particular case: it is the invention of a person of the name of Coles, residing at London-bridge; and, although it is not very easy to convey an accurate idea of any instrument by mere words, we may perhaps be able to afford some explanation of the difference which exists between this and the common truss, by remarking that two spiral springs are introduced between the truss and the pad, which, whilst it admits of a self-adjusting movement, permits a certain degree of yielding pressure. We are acquainted with several cases in which these trusses are now made use of, and the principle appears to us sufficiently novel and ingenious to induce us to draw the attention of the medical profession to investigate its merits.

### OBITUARY.

### METEOROLOGICAL JOURNAL.

By Messrs. William Harris and Co. 50, Holborn, London.

*From December* 20, 1822, *to January* 19, 1823.

| | MOON. | Rain gauge | THERM. | | | BAROM. | | DE LUC'S HYG. | | WIND. | | ATMOSPHERIC VARIATION. | | |
|---|---|---|---|---|---|---|---|---|---|---|---|---|---|---|
| | | | 9 A.M. | MAX. | MIN. | 9 A.M. | 10P.M. | 9 A.M. | 10P.M. | 9 A.M. | 10P.M. | 9 A.M. | 2 P.M. | 10P.M. |
| Dec | | | | | | | | | | | | | | |
| 20 | | | 28 | 34 | 26 | 30.18 | 30.16 | 71 | 73 | E | E | Fine | | |
| 21 | ☾ | | 27 | 34 | 29 | 30.15 | 30.09 | 75 | 72 | E | E | Fine | | |
| 22 | | | 34 | 38 | 33 | 30.03 | 29.95 | 74 | 71 | E | ENE | Fine | | |
| 23 | | | 38 | 39 | 34 | 29.90 | 29.87 | 74 | 85 | E | E | Fine | Fair | Sleet |
| 24 | | | 38 | 34 | 31 | 29.88 | 30.00 | 82 | 75 | E | SE | Cloudy | Fine | |
| 25 | | | 34 | 34 | 27 | 30.26 | 30.29 | 65 | 74 | ESE | E | Fine | | |
| 26 | | | 30 | 31 | 28 | 30.27 | 30.25 | 81 | 75 | E | ESE | Fine | | |
| 27 | | | 30 | 34 | 21 | 30.17 | 30.30 | 81 | 79 | ESE | SE | Foggy | Fair | Fine |
| 28 | ○ | | 23 | 31 | 22 | 30.12 | 30.04 | 77 | 71 | SE | SE | Fine | | |
| 29 | | | 30 | 30 | 20 | 29.95 | 29.86 | 80 | 72 | SSE | SE | Fine | | V.Fine |
| 30 | | | 24 | 30 | 26 | 29.70 | 29.62 | 72 | 70 | SE | ESE | Fair | Fine | |
| 31 | | | 28 | 30 | 28 | 29.59 | 29.56 | 68 | 74 | ESE | SE | Fair | Snowy | Fair |
| Jan. | | | | | | | | | | | | | | |
| 1 | | | 29 | 38 | 28 | 29.60 | 29.60 | 77 | 80 | SW | SSE | Foggy | Overc. | Fair |
| 2 | | | 30 | 41 | 38 | 29.63 | 29.71 | 90 | 74 | S var. | SE | Rain | Cloud. | |
| 3 | | | 41 | 46 | 40 | 29.68 | 29.61 | 81 | 75 | SE | SE | Cloudy | Rain | Fair |
| 4 | ☽ | | 40 | 48 | 37 | 29.58 | 29.59 | 77 | 81 | SE | SE | Fair | Cloud. | Rain |
| 5 | | | 38 | 42 | 39 | 29.54 | 29.57 | 95 | 96 | SE | SSW | Rain | Sho'ry | Rain |
| 6 | | | 44 | 46 | 35 | 29.72 | 29.90 | 97 | 95 | SSW | SE | Cloudy | | |
| 7 | | | 37 | 42 | 33 | 29.96 | 30.02 | 87 | 85 | ESE | E | Cloudy | Fine | |
| 8 | | | 34 | 38 | 27 | 30.03 | 29.96 | 86 | 90 | E | E | Cloudy | Fine | |
| 9 | | Rain guage frozen. | 30 | 35 | 27 | 29.87 | 29.77 | 87 | 82 | E | ESE | Cloudy | Fine | |
| 10 | | | 32 | 34 | 26 | 29.77 | 29.69 | 81 | 87 | E | E | Cloudy | | |
| 11 | | | 26 | 36 | 23 | 29.73 | 29.79 | 80 | 75 | E | E | Fine | | |
| 12 | ● | | 24 | 30 | 24 | 29.61 | 29.67 | 71 | 75 | ENE | NNE | Fair | Fine | Sleet |
| 13 | | | 26 | 30 | 21 | 29.55 | 29.51 | 80 | 70 | ENE | NW | Snow | Fair | Foggy |
| 14 | | | 22 | 30 | 22 | 29.43 | 29.58 | 76 | 75 | SE | SE | Foggy | Fine | Foggy |
| 15 | | | 23 | 30 | 24 | 29.12 | 29.18 | 86 | 89 | NE | NE | Snow | Fine | Fair |
| 16 | | | 26 | 30 | 28 | 29.22 | 29.22 | 90 | 85 | N | NNW | Cloudy | | |
| 17 | | | 28 | 30 | 27 | 29.25 | 29.25 | 87 | 82 | NNE | NW | Cloudy | | |
| 18 | | | 30 | 31 | 16 | 29.23 | 29.27 | 85 | 86 | NW | N | Cloudy | Fine | Cloud. |
| 19 | | | 17 | 21 | 12 | 29.31 | 29.44 | 85 | 87 | NNW | ENE | Yel.Dew Fog | Foggy | |

The quantity of rain during the month of December, 1822, was 1 inch and 40.100ths.

